# Azathioprine‐induced pellagra in a child with autoimmune hepatitis: A case report and literature review

**DOI:** 10.1002/ccr3.4975

**Published:** 2021-10-17

**Authors:** Bahareh Abtahi‐Naeini, Parvin Rajabi, Shakiba Dehghani

**Affiliations:** ^1^ Pediatric Dermatology Division of Department of Pediatrics Imam Hossein Children's Hospital, Isfahan University of Medical Sciences Isfahan Iran; ^2^ Skin Diseases and Leishmaniasis Research Center Isfahan University of Medical Sciences Isfahan Iran; ^3^ Department of Pathology Isfahan University of Medical Sciences Isfahan Iran; ^4^ School of medicine Isfahan University of Medical Sciences Isfahan Iran

**Keywords:** autoimmune hepatitis, azathioprine, drug eruptions, pellagra, vitamin B deficiency

## Abstract

Pellagra is a clinical syndrome resulting from niacin deficiency with variety of manifestations. Azathioprine is among drugs that can lead to such condition. Physicians should be aware as proper management can lead to full resolution.

## Graphical abstract

Pellagra is a clinical syndrome resulting from niacin deficiency with variety of manifestations. Azathioprine is among the drugs that can lead to such condition. Physicians should be aware as proper management can lead to full resolution.

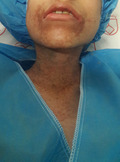

## 1 CASE PRESENTATION

**FIGURE 1:**
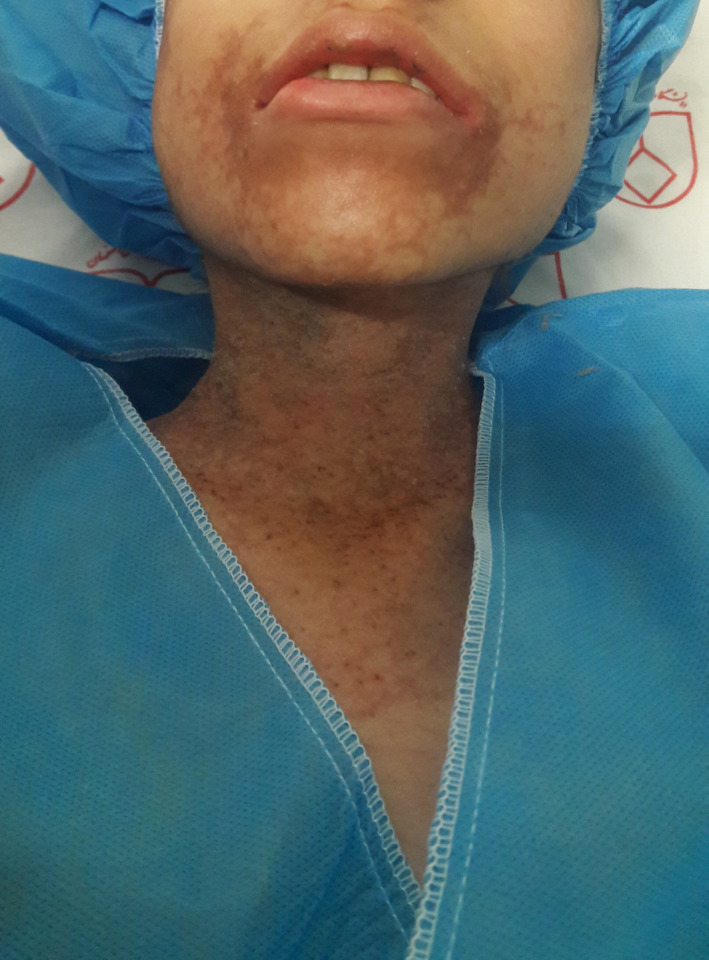
Azathioprine‐induced pellagra. Well‐demarcated violaceous to brown skin lesion on the neck (Casal's collar) and periorificial area

**FIGURE 2:**
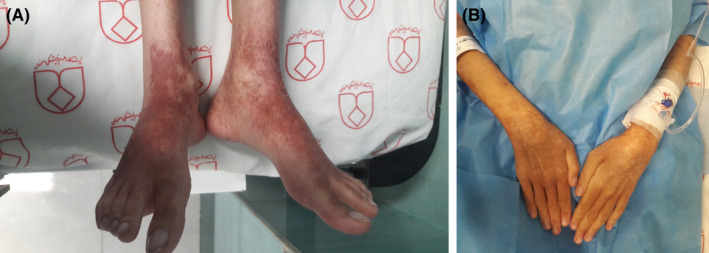
Azathioprine‐induced pellagra. Dorsal foot (A) and dorsal hand and forearm (B) show dry, cracked, and hyperpigmented skin lesions associated with desquamation

Pellagra, a potentially fatal but easily treated disorder, is characterized by symmetrical photo‐distributed skin lesions due to niacin deficiency. There are many known etiologies for pellagra including drug‐induced cases. We, hereby, present a new case of azathioprine‐induced pellagra and briefly review reported cases of azathioprine‐induced pellagra in the literature. A 14‐year‐old girl was admitted with a 3‐day history of abdominal pain and diarrhea. She had a recent diagnosis of autoimmune hepatitis 8 months ago and azathioprine (AZA) was initiated 4 weeks before. She was underweight (body mass index of 17.7 kg/m^2^). Further history revealed poor nutritional intake due to decreased appetite, but she denied any specific dietary restrictions. The patient also complained of a 1‐day history of new onset painful skin lesions. She had no personal or family history of skin diseases. Well‐demarcated violaceous to brown skin lesions with scaly desquamation and hyperpigmentation were present on the neck, dorsum of the hands, and feet. The patient reported that the rash developed shortly after having sun exposure in the preceding days (Figures 1 and 2).

Based on the photo distribution of dermatitis and associated scaling and hyperpigmentation in the pattern of casal's necklace in association with the recent initiation of AZA, a clinical diagnosis of AZA‐induced pellagra was made. Our patient's total Naranjo Scale score was 6 (possible adverse drug reaction).

**FIGURE 3:**
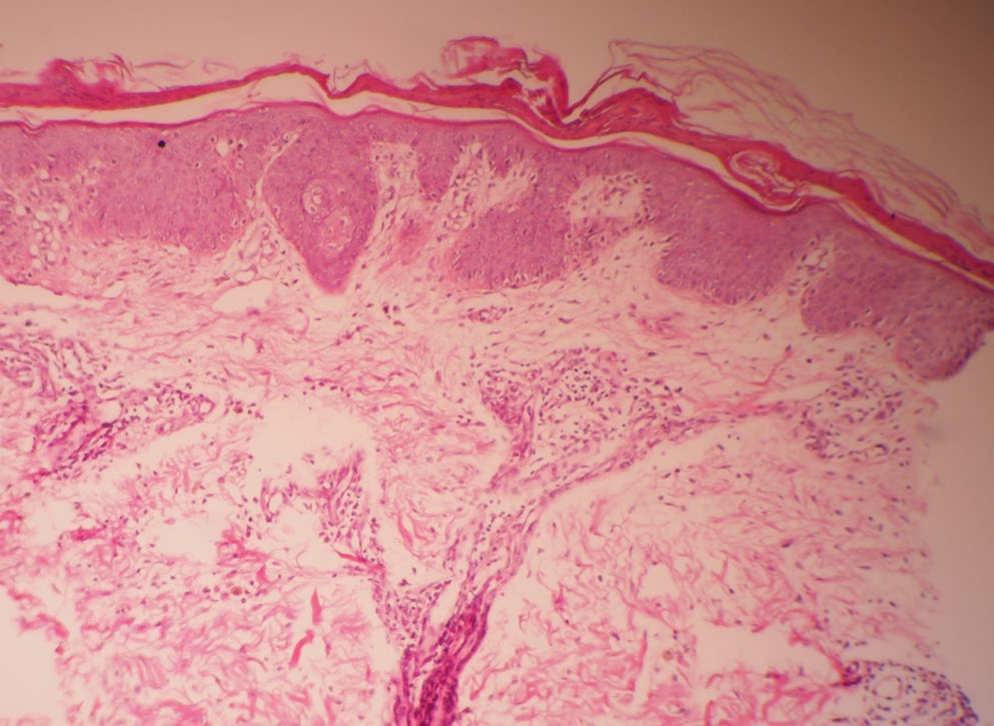
Histopathological images of Azathioprine‐induced pellagra. (HE, hematoxylin‐eosin, ×100.) Histopathological examination showed mild acanthosis, confluent parakeratosis in the epidermis with regular elongation of rete ridges. In the epidermis, there is superficial perivascular infiltration

Histopathological examination showed confluent parakeratosis, mild acanthosis, and regular elongation of rete ridges. There was focal subepidermal blistering with dermal capillary proliferation and a mild perivascular infiltrate (Figure 3).

Discontinuation of azathioprine and administration of oral nicotinamide 50 mg twice daily resulted in significant improvement of the rash within 2 days. The dosage of nicotinamide was gradually increased over the following 4 weeks. After 2 months of treatment, only mild postinflammatory hyperpigmentation remained.

## 2 DISCUSSION

Pellagra is a clinical syndrome resulting from niacin deficiency. It is an underdiagnosed but still existing disease. Pellagra is clinically characterized by the classic triad of 3 D's: (1) dermatitis (photo‐distributed symmetrical skin lesions), (2) diarrhea, and (3) dementia (neurologic and psychotic disturbances) which can ultimately lead to death if left undiagnosed or untreated.^1^

Histological features of pellagra are perivascular lymphocytic infiltrate in the upper dermis and edema in the papillary dermis which can be observed in the acute stages. Hyperkeratosis, parakeratosis, and epidermal atrophy are mostly seen in late stages. Although these features are unspecific, they can support the clinical diagnosis.^2^

The main cause of pellagra is niacin or tryptophan (niacin precursor) deficiency. Pellagra has been reported to be associated with some medications including isoniazid, 6‐mercaptopurine (6‐MP), 5‐fluorouracil, and also azathioprine (AZA).^1^

AZA is metabolized to 6‐MP. 6‐MP decreases the synthesis of nicotinamide adenine dinucleotide and nicotinamide adenine dinucleotide phosphate, which are key coenzymes in niacin metabolism and other metabolic pathways. Consequently, lack of these coenzymes will result in major dysfunction in tissues with high energy demands such as brain, gut, and skin.^3^, ^4^, ^5^

Thus, it is likely that AZA can lead to secondary niacin deficiency and development of pellagra.

In our patient, underlying poor nutrition as a result of her chronic disease may have caused a relative niacin deficiency that was exacerbated by addition of azathioprine, culminating in the clinical presentation.

AZA‐induced pellagra has been rarely reported, predominantly in adults.^2^, ^3^, ^5^ Herein, we briefly review previous AZA‐induced pellagra cases reported in the literature. (Table 1).

**TABLE 1:** Previous Azathioprine‐induced pellagra reported in the literature

Number	Author/year	Patient age/sex	Preexisting disease	Duration of azathioprine use	Dermatologic manifestations	Other clinical manifestations	treatment	Prognosis
1	Jarrett et al. (1997)^2^	42/female	Ulcerative colitis	2 weeks	Browny‐red lesions with peeling and distinct margin resembling sunburn on photo‐exposed areas affecting both hands, the radial side of forearms, uncovered parts of feet, casal's necklace.	No mental confusion	Azathioprine was not discontinued, Nicotinamide 500 mg once daily.	Rash quickly improved and resolved
2	Jarrett et al. (1997)^2^	17/female	Crohn's disease	10 days	Marginated, reddish‐brown, scaling rash extending down the radial aspects of forearms from just above elbows, casal's necklace, more erythematous on the dorsa of the hands, the front and backs of the legs, and the dorsa of the feet.	No mental confusion	Azathioprine discontinued, nicotinamide 50 mg three times daily.	No resolution until azathioprine discontinuation. Rapid improvement and full resolution after starting nicotinamide
3	Oliveira et al. (2011)^5^	47/female	Polymyositis	15 years	Painful, well‐defined, erythematous, arched plaque with exudative surface on anterior cervical region appearing after sun exposure. Multiple erosions on dorsum of the hand recovered by sero‐hematic crusts.	Acute onset diarrhea, chelitis, glossitis	Azathioprine was discontinued, 300 mg niacin/day/oral began.	As skin lesions were improving, patient developed medullary aplasia leading to death three weeks later.
4	Zhao et al. (2018)^4^	50/female	Neuromyelitis optica	3 months	Multiple painful erythematous, well‐defined plaques on the dorsum of hands spreading to wrists, with scaling.	Multiple tongue and buccal mucus ulcerations, hyperpathia in thorax and abdomen, thoracic and abdominal pain, paroxysmal girdle‐like tightening sensation leading to depression	Azathioprine was discontinued, nicotinamide 100 mg 3 times daily +vitamin B complex three times daily	Rapid improvement of skin lesions, within 1 month fully resolved skin lesions and no hyperpathia or zonesthesia
5	Present case	14/female	Autoimmune hepatitis	4 weeks	Well‐demarcated violaceous to brown skin lesions with scaly desquamation and hyperpigmentation on the neck, dorsum of the hands, and feet which aggravated after sun exposure	Diarrhea, abdominal pain	Azathioprine was discontinued, oral nicotinamide 50 mg twice daily initiated.	Significant improvement after nicotinamide administration, full resolution after 2 months of treatment

To the best of our knowledge, there are only four cases of AZA‐induced pellagra reported in the literature. All the reported cases were female patients, and only one case was in pediatrics. Duration of AZA use varied widely among patients from days to years. They all had the typical skin manifestations of pellagra, some experienced diarrhea, and none had neurologic disturbance. AZA was discontinued in all the patients except one, and they were all initiated on nicotinamide with different dosages from 150 to 500 mg/day. Significant improvement in skin lesions was seen in all the cases.^2^, ^3^, ^5^

Clinicians should consider AZA‐induced pellagra in any patient who develops a photo‐distributed dermatosis while undergoing treatment with AZA.

## CONFLICT OF INTEREST

The authors declare that there is no conflict of interests regarding the publication of this paper.

## AUTHOR CONTRIBUTIONS

Bahareh Abtahi‐Naeini had contributed to designing and conducting the study. Parvin Rajabi had contributed to pathological reports of the study. Shakiba Dehghani had assisted in the preparation of the first draft of the manuscript and manuscript revision. All authors have revised the manuscript critically for important intellectual content, also have read and approved the content of the manuscript, and confirmed the accuracy or integrity of any part of the work.

## CONSENT

Written informed consents were obtained from the patient's guardians for publication of this paper and any accompanying images.

## Data Availability

The data that support the findings of this study are available from the corresponding author upon reasonable request.
